# Histone Modification‐Dependent Transcriptional Regulation of Defence Genes in Early Response of Arabidopsis to *Spodoptera litura* Attack

**DOI:** 10.1111/pce.15345

**Published:** 2024-12-25

**Authors:** Ahmed Yusuf, Kota Wakaya, Takuya Sakamoto, Takuya Uemura, Koudai Okamura, Abdelaziz Ramadan, Akira Nozawa, Takamasa Suzuki, Yayoi Inui, Sachihiro Matsunaga, Tatsuya Sawasaki, Gen‐Ichiro Arimura

**Affiliations:** ^1^ Department of Biological Science and Technology Faculty of Advanced Engineering, Tokyo University of Science Tokyo Japan; ^2^ Department of Botany Faculty of Science, Ain Shams University Cairo Egypt; ^3^ Department of Science Faculty of Science, Kanagawa University Yokohama Japan; ^4^ Proteo‐Science Center Ehime University Matsuyama Japan; ^5^ College of Bioscience and Biotechnology Chubu University Kasugai Japan; ^6^ Department of Integrated Biosciences Graduate School of Frontier Sciences, The University of Tokyo Kashiwa Japan

**Keywords:** *Arabidopsis thaliana*, histone acetyltransferase of the CBP family 1 (HAC1), histone deacetylase 6 (HDA6), *Spodoptera litura*, TOPLESS (TPL) and TOPLESS‐related (TPR) corepressors

## Abstract

Histone modification is a cellular process for transcriptional regulation. In herbivore‐damaged plants, activation of genes involved in defence responses is required for antiherbivore properties, but little is known about how the chromatin remodelling system is involved. In Arabidopsis (*Arabidopsis thaliana*) plants responding to *Spodoptera litura* larvae, HAC1 and HDA6, a histone acetyltransferase and a histone deacetylase, respectively, were found here to be involved in histone H3 (Lys9; H3K9) acetylation/deacetylation at the promoter region of the plant defensin gene *PDF1.2* and the gene body of ethylene response factor 13 (*ERF13*) as early as 2 h after the onset of herbivore attack. The H3K9 acetylation was responsible for the robust upregulation of *PDF1.2* later, at 24 h, and *ERF13* even earlier, at 1 h. TOPLESS (TPL) and TOPLESS‐related (TPR) corepressors interacted with HDA6 to deacetylate H3K9 at *PDF1.2* and *ERF13*, while negatively regulating the expression of *PDF1.2* but not *ERF13*. Furthermore, TPL also interacted with ERF13, resulting in ERF13‐mediated regulation of *PDF1.2*. Taken together, these data suggest a model of promoter‐restricted, TPL/TPR‐directed histone deacetylation and transcription factor repression in healthy Arabidopsis plants for the feedback regulation of the antiherbivore response.

## Summary statement

The early timing of defence gene activation after the onset of herbivore damage is regulated by histone modifications that are highly dependent on specific histone acetyltransferases/deacetylases (HAC1/HDA6) with TPL/TPR corepressors in *Arabidopsis thaliana*.

## 1 Introduction

Plants have evolved molecular mechanisms to effectively protect themselves against a variety of threats, such as herbivores. These defence mechanisms rely primarily on the regulation of genes responsible for defence‐related proteins and specialized compounds, such as toxins and infochemicals (Okada, Abe, and Arimura 2015). Gene regulation plays a critical role in determining when and where these defence genes are expressed, and this intricate process involves several mechanisms, including the action of regulatory proteins known as transcription factors (Singh, Foley, and Onate‐Sanchez 2002). Importantly, the control of defence gene expression may also involve epigenetic regulation, specifically through histone modifications and DNA methylation (Rasmann et al. 2012; Wilkinson et al. 2023).

In this context, *Arabidopsis thaliana* (Arabidopsis) plants exhibit an induced defence response against herbivores when exposed to β‐ocimene, a volatile herbivore danger signal, emitted by neighbouring plants damaged by herbivores. This response is associated with increased histone acetylation and transcriptional activity of defence genes, particularly ethylene response factor genes (*ERF8* and *ERF104*) in leaves (Onosato et al. 2022). The increased defence capacity of these plants is observed up to 5 days after β‐ocimene exposure and coincides with the upregulation of *ERFs*. This positive regulation of *ERFs* is mediated by histone acetyltransferases (HATs) such as histone acetyltransferase of the CBP family 1 (HAC1), while other HATs such as HAC5 and HAM1 are also partially involved in the histone acetylation regulation at *ERFs* in β‐ocimene‐exposed leaves. In particular, HAC1 is likely to act as a master regulator of both histone H3 and H4 acetylation at these two *ERFs*.

HAC1, which possesses H3K9 and H4K14 acetyltransferase activities, plays a dominant role in plant defence responses, including those induced by salt stress, UV‐B and pathogen infection (Fina et al. 2017; Li et al. 2014; Singh et al. 2014; Zheng et al. 2019). HAC1 specifically acetylates histones associated with target genes of the jasmonate‐associated transcription factor MYC2 and promotes their expression by interacting with MED25, a component of the mediator complex involved in transcriptional regulation (An et al. 2017). The HAC1/MED25 system was found to increase transcription by acetylating the *ERF022* promoter during the age‐related senescence of Arabidopsis leaves (Hinckley et al. 2019).

In addition, the involvement of histone deacetylases (HDACs) in chromatin remodelling is important for the feedback regulation of gene expression (Jiang et al. 2020). Among these HDACs, HDAC6 (HDA6) stands out because it specifically removes acetyl groups from lysine residues in the histone H3 and H4 N‐tails, excluding H4K16 (To et al. 2011). By repressing defence responses during pathogen infection, HDA6, plays a critical role in inhibiting and modulating the expression of pathogen‐responsive genes in Arabidopsis (Wang et al. 2013). Notably, HDA6 also acts as a repressor of both jasmonate and ethylene signalling (Wu et al. 2008; Zhu et al. 2011); thus, it is conceivable that HDA6 may act as a key factor in the chromatin remodelling system of plants damaged by herbivores, such as *Spodoptera* spp., where jasmonate and ethylene play roles in cellular signalling (Arimura 2021). However, there is no proof of this yet.

In this study, our primary objective was to understand the epigenetic transcriptional regulation system in Arabidopsis plants in response to damage by larvae of *Spodoptera litura*, a generalist herbivore that causes significant damage to agricultural crops worldwide (Nagoshi, Brambila, and Meagher 2011). Our working model centred on the critical roles of HAC1 and HDA6 in facilitating histone remodelling during plant defence against herbivores. To test this model, we performed an in‐depth gene mining analysis to identify the genes controlled by these factors in Arabidopsis plants when infested with *S. litura* larvae. Our results showed that these factors regulate histone modifications as early as 2 h after feeding, leading to subsequent gene upregulation. Furthermore, the involvement of the TOPLESS (TPL) and TOPLESS‐related (TPR) corepressors, which cofunction with HDA6 in Arabidopsis plants during herbivory, was addressed, further enhancing our understanding of these complex mechanisms.

## 2 Materials and Methods

### 2.1 Plants

*A. thaliana* (Arabidopsis) ecotype Col‐0, T‐DNA insertion mutant line of Col‐0 (*hac1* [SALK_080380]), *hda6* (*axe1‐4* [Murfett et al. 2001]) and *tpl*/*tpr1*/*tpr4* (Zhu et al. 2010), and transgenic lines of Col‐0 (see below) were grown for 4 weeks in a growth chamber at 22 ± 1°C with a photoperiod of 12 h (80 µE m^–2^ s^–1^). *Nicotiana benthamiana* plants were grown at 24 ± 1°C with a photoperiod of 16 h. Individual plants were grown in individual plastic pots.

### 2.2 Primers

Primers used for polymerase chain reaction (PCR) are listed in Supporting Information S1: Table S1.

### 2.3 Generation of Transgenic Plants

The *HDA6* genomic region or the open reading frame (ORF) of each of the *TPL/TPR* cDNAs was inserted into the Gateway (GW) vector pMDC32 (2x cauliflower mosaic virus 35S promoter [35SP]::GW:: *nopaline synthase* terminator [NOST]) using the GW cloning system, to produce the resulting vectors: pMDC32‐HDA6, pMDC32‐TPL, pMDC32‐TPR1, pMDC32‐TPR2, pMDC32‐TPR3 and pMDC32‐TPR4. These vectors were transformed into *Agrobacterium tumefaciens* strain EHA105 by electroporation. Wild‐type (WT) Arabidopsis plants that had been grown for approximately 6–7 weeks were transformed by the floral‐dip transformation method (Clough and Bent 1998). T1 transgenic seeds from each transformant were tested for germination on 1/2 MS medium supplemented with 30 mg L^–1^ hygromycin. T2 seeds, which showed a segregation ratio of approximately 3:1, were again tested for hygromycin resistance. T3 homozygous plant lines were used for further analysis.

### 2.4 S. litura

Eggs of *S. litura* (Fabricius) were obtained from Sumika Technoservice Co. Ltd. (Takarazuka, Japan). They were incubated in an air‐conditioned room at 24 ± 1°C with a photoperiod of 16 h according to the methods as described previously (Uemura et al. 2020). For treatment of plants with *S. litura*, five third instar larvae were released on a potted plant in the laboratory at 24 ± 1°C with a photoperiod of 16 h for up to 24 h, except for the assays in Supporting Information S1: Figure S7 where 20 third instar larvae were released on a potted plant. The visually damaged leaves were collected and used for analysis.

### 2.5 Collection and Processing of Oral Secretion (OS)

OS was collected from third or fourth instar *S. litura* larvae (approximately 1 week after hatching) using a glass capillary tube (Hirschmann Laborgeräte GmbH and Co. KG, Eberstadt, Germany). The collected OS was stored at –20°C until use. Mechanical damage (MD) was performed using stainless steel needles on 6−8 leaves of a single potted Arabidopsis plant. Approximately 40 MD spots were made per leaf. OS was immediately applied to an MD spot (approximately 1 µL per spot).

### 2.6 Chromatin Immunoprecipitation (ChIP)‐Sequencing (ChIP‐Seq)

ChIP was performed with modifications from Luo and Lam (2014). Approximately 1.5 g of leaf tissue was frozen in liquid nitrogen, ground to a fine powder, crosslinked, nuclear extracted and sonicated according to the method of Onosato et al. (2022). The sonicated chromatin sample was divided into equal amounts according to the number of antibodies used. The following antibodies were used: anti‐acetyl histone H3K9 (MABI0305; FUJIFILM Wako Pure Chemical Corporation, Osaka, Japan) and rabbit anti‐H3 (ab1791; Abcam, Tokyo, Japan). Immunoprecipitation, washing, reverse cross‐linking and DNA extraction were performed as described previously (Kimura et al. 2008), and 1 ng of DNA was used to construct a library for Illumina sequencing. The library was prepared using the KAPA Hyper Prep Kit for Illumina (KAPA Biosystems, Wilmington, USA) and double size selected using Agencourt AMPure XP (Beckman Coulter, Indianapolis, IN, USA) to enrich for 300−500 bp fragments. DNA libraries were sequenced using a NextSeq500 (Illumina, San Diego, CA, USA) in single‐end mode or a HiSeq X Ten (Illumina) in paired‐end mode. Reads were mapped to the Arabidopsis reference using Bowtie 2 (Langmead et al. 2009). To report only uniquely mapped reads, the multiple mapped reads were excluded from the resulting SAM files using the following command: ‘grep ‐v “XS:”’. The SAM files were converted to sorted BAM files using SAMtools (Li et al. 2009). Enrichment peaks of H3K9 acetylation (H3K9ac) (*q* < 0.05) were retrieved by using MACS2 (Zhang et al. 2008) with H3 as the baseline control and with options of ‘‐‐extsize 147’ and ‘‐g 1.3e8’. The bdgdiff function of MACS2 was used to identify differentially enriched peaks of H3K9ac between two samples with histone H3 as the control, and these identified peaks were assigned to genes using ChIPseeker (Yu, Wang, and He 2015) with the default setting. The analyses of H3K9ac peaks were performed using the second replicate. BAM files were also converted into BED files using BEDTools (Quinlan and Hall 2010). The ‘slop’ function of BEDTools was used to extend the 5′ end of the ChIP‐seq reads in the 3′ direction to match the average insertion size (300 bp) of the sequenced libraries. The coverage function of BEDTools was then used to calculate the number of reads that overlapped with each annotation unit. Then, the level of H3K9ac at each gene was calculated as follows: reads per million mapped reads (RPM) of acetylated histone H3K9/RPM of histone H3. The average RPM of two replicates was used for this calculation. All sequence data is registered in the DDBJ Sequence Read Archive (PRJDB18711).

To display the distribution of H3K9ac at the genes of interest, the output BAM files were converted to TDF files with default settings and visualized by using IGV software. PlantRegMap (https://plantregmap.gao-lab.org/go_result.php) was used for the gene ontology (GO) analysis.

### 2.7 ChIP and Quantitative PCR (qPCR) Analysis

ChIP‐qPCR was performed as described by Onosato et al. (2022) with minor modifications using anti‐acetyl histone H3K9 (see above) or anti‐HDA6 (PHY1809A; PHYTOAB, San Jose, CA, USA) coupled to protein G magnetic beads (MEDICAL & BIOLOGICAL LABORATORIES Co. Ltd., JSR Life Science, Nagoya, Japan). Precipitated DNA fragments were quantified by qPCR (see below), and relative abundances were determined after normalization of their input control, raw signals to those of DNA fragments without precipitation.

### 2.8 RNA Extraction, cDNA Synthesis and qPCR

Approximately 100 mg of leaf tissues were homogenized in liquid nitrogen, and total RNA was isolated and purified using TRI‐REAGENT‐RNA/DNA/Protein isolation reagent (Molecular Research Center Inc., Cincinnati, OH, USA) following the manufacturer's protocol. First‐strand cDNA was synthesized using ReverTra Ace qPCR RT Master Mix with gDNA Remover (Toyobo, Osaka, Japan) and 0.5 µg of total RNA incubated first at 37°C for 5 min for the DNase reaction and second at 37°C for 15 min for the reverse transcriptase reaction. Real‐time PCR was performed using a CFX Connect Real*‐*Time PCR detection system (Bio‐Rad, Hercules, CA, USA) with THUNDERBIRD SYBR qPCR Mix (Toyobo) and gene‐specific primers (Supporting Information S1: Table S1). The following protocol was used: initial polymerase activation: 60 s at 95°C; 40 cycles of 15 s at 95°C and 30 s at 60°C; and then melting curve analysis preset by the instrument was performed. Relative expression abundances were determined after normalization of raw signals with the expression abundance of a housekeeping *ACT8* gene. We did not use samples or data when abnormal quantification cycle (Cq) values were obtained.

### 2.9 Cell‐Free Protein Synthesis, Immunoblotting and AlphaScreen System

The full‐length ORFs of *GFP* (green fluorescent protein serving as control), *TPL*, *TPR1*, *TPR2*, *TPR3* and *TPR4* were inserted into the GW vector pEU‐FLAG‐GW. Similarly, the full‐length ORF of *HDA6* (without stop codon) was inserted into pEU‐GW‐6 × His‐bls (bls; biotin ligation site). Cell‐free protein synthesis and AlphaScreen‐based protein−protein interaction assays were carried out according to the methods described previously (Yano et al. 2016).

To evaluate the quality of the proteins used, total proteins were subjected to 10% sodium dodecyl sulfate (SDS)‐polyacrylamide gel electrophoresis and immunoblotted with anti‐biotin HRP‐conjugated antibody (Cell Signalling Technology, Beverly, MA, USA) or monoclonal anti‐FLAG M2 peroxidase antibody produced by mouse clone M2 (Sigma‐Aldrich, St. Louis, MO, USA). The membranes were immobilized with Immobilon Western Chemiluminescent HRP Substrate (Merck Millipore Ltd., Darmstadt, Germany) and signals were detected using an ImageQuant LAS‐4000 imaging system (GE Healthcare, Buckinghamshire, UK) (Supporting Information S1: Figure S1).

### 2.10 Bimolecular Fluorescence Complementation (BiFC) Analysis

The full‐length ORF of *HDA6* or *ERF13* (without stop codon) was inserted into pDEST‐GW‐nVenus (35SP::GW::N‐terminal fragment of Venus [nVenus]::NOST), resulting in the construction of the vector HDA6‐nVenus or ERF13‐nVenus. Similarly, the full‐length ORFs of *TPL*/*TPR* (*TPL*, *TPR1, TPR2, TPR3* or *TPR4*; without stop codon) were inserted into pDEST‐GW‐cVenus (35SP::GW::C‐terminal fragment of Venus::NOST), resulting in the construction of vectors TPL/TPR‐cVenus. These vectors were transformed into *A. tumefaciens* strain EHA105 by electroporation.

A pair of *A. tumefaciens* cell suspensions carrying the individual vectors (HDA6‐nVenus and each of TPL/TPR‐cVenus or ERF13‐nVenus and each of TPL/TPR‐cVenus) was pressure‐infiltrated into the leaves of *N. benthamiana* plants. The transformed plants were incubated for 48 h in an air‐conditioned room at 24 ± 1°C, and the fluorescence was observed under a BZ‐X700 fluorescence microscope (Keyence Co., Osaka, Japan). For nuclear staining, the sample was pretreated with 0.2 mM 4',6‐diamidino‐2‐phenylindole (DAPI) (FUJIFILM Wako Pure Chemical Corporation Ltd.) for 5 min before observation.

### 2.11 Foliage Damage Assessment by *S. litura* Larvae

Three third instar larvae of *S. litura* were placed on a potted plant. After 24 h of incubation in the laboratory at 24 ± 1°C with a 16 h photoperiod, the leaves were scanned and the exact area consumed by the larvae was measured using ImageJ.

### 2.12 Protoplast Preparation, Transfection and Luciferase (LUC) Assay

The full‐length ORF of *ERF13* was cloned into the p35SΩ‐GW‐V5‐NOST vector (35SP::Ω sequence [translation enhancer]::GW::V5::NOST), resulting in the construction of the vector p35SΩ‐ERF13‐V5‐NOST. This plasmid was transformed into the leaf mesophyll protoplasts with the reporter vector in which the promoter region (40−1000 bp upstream region) of *PDF1.2* (PDF1.2 P) was inserted in front of the firefly luciferase (Fluc) reporter gene::NOST cassette in the pMA cloning vector (pMA‐PDF1.2P‐Fluc‐NOST) (Kawaguchi et al. 2023) and reference (35SP::Renilla luciferase [Rluc]::NOST [p35SP‐Rluc‐NOST]) vector.

Protoplasts were isolated from approximately 50 mature Arabidopsis WT, TPL‐OX2 and *tpl*/*tpr1*/*tpr4* leaves by the tape Arabidopsis‐sandwich method (Wu et al. 2008). Finally, 2.0 × 10^5^ protoplasts mL^−1^ were prepared. Polyethylene glycol (PEG)‐mediated DNA transfection was performed as previously described (Yoo, Cho, and Sheen 2007). The protoplast suspension (2.0 × 10^4^ protoplasts in 100 µL) was supplemented with a mixture of vectors carrying pMA‐PDF1.2‐Fluc‐NOST with p35SP‐Rluc‐NOST, additionally with or without p35SΩ‐ERF13‐V5‐NOST at a ratio of 4:1:(5) to protoplast suspension with 100 µL of PEG solution (40% [w/v] polyethylene glycol, 0.6 M mannitol and 15 mM Ca[NO_3_]_2_·4 H_2_O). The transfection was carried out at room temperature for 15 min and stopped by adding 400 µL of W5 solution (2 mM 2‐morpholinoethanesulfonic acid, monohydrate [MES] [pH 5.7], 12.5 mM CaCl_2_, 154 mM NaCl and 5 mM KCl). The protoplasts were collected by centrifugation at 200*g* for 2 min and resuspended with 500 µL of WI solution (5 mM MES [pH 5.7], 0.6 M mannitol and 20 mM KCl) and incubated at room temperature overnight.

The LUC assay was performed as previously described (Miyamoto et al. 2019) with a minor modification. Fluc activity produced due to the transfected reporter construct was expressed as the value normalized by the Rluc activity produced due to the cotransfected reference vector.

### 2.13 Statistics and Reproducibility

We performed Student's *t*‐test for pairwise analyses and one‐way analysis of variance (ANOVA) with Holm's sequential Bonferroni post hoc test or Tukey's post hoc honestly significant difference (HSD) for multiple‐sample analyses using the multiple‐sample comparison program (http://astatsa.com/OneWay_Anova_with_TukeyHSD/). Sample sizes and number of replicates for all sets of assays and analyses are indicated in the legends of the corresponding figures.

## 3 Results

### 3.1 Enriched Histone Acetylation in Leaves in Response to *S. litura* Attack

**Figure 1:**
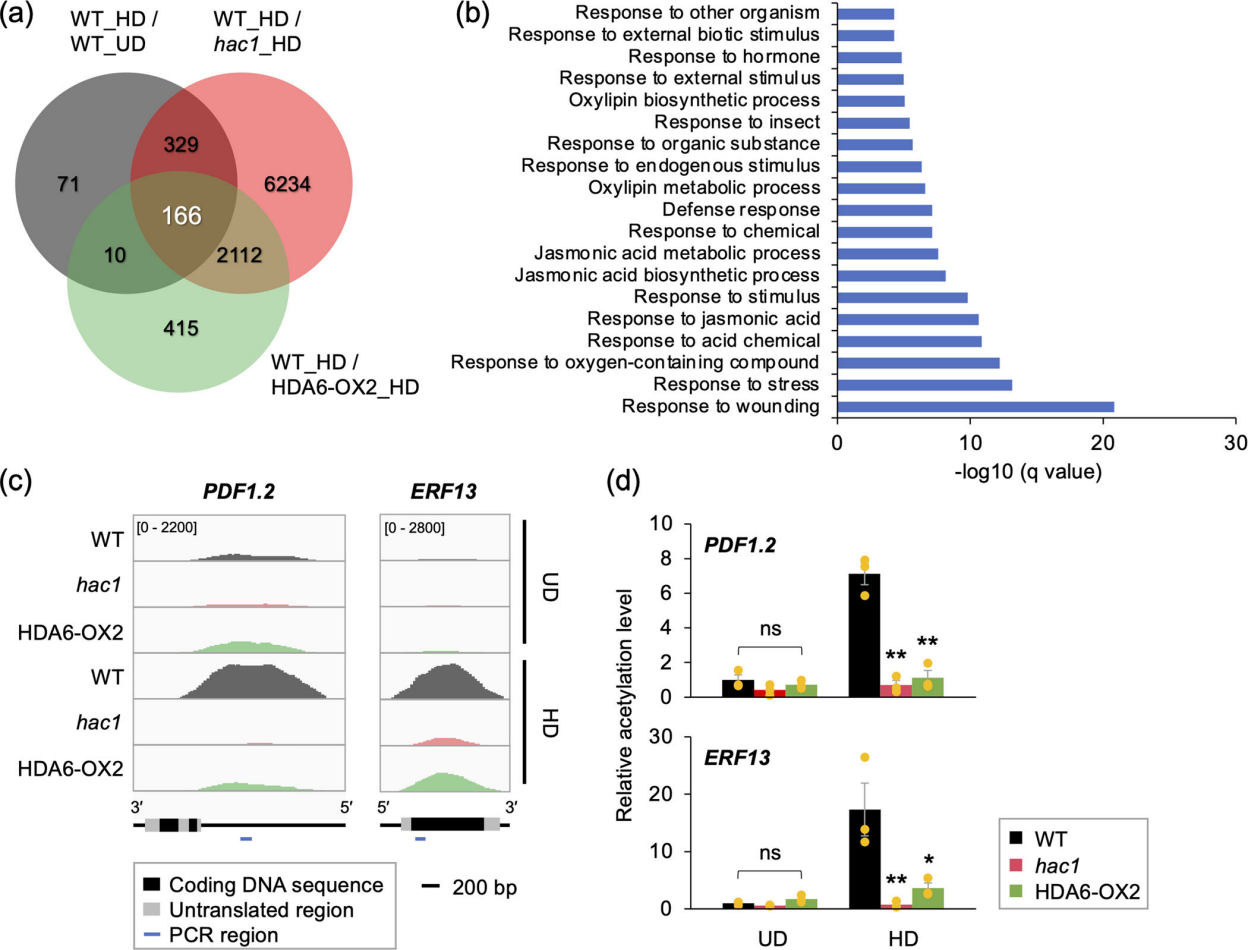
Changes in H3K9 acetylation (H3K9ac) levels in leaves of wild‐type (WT), *hac1* mutant and HDA6‐overexpressing (HDA6‐OX2) plants before and after damage by *Spodoptera litura* larvae. (a) Venn diagram illustrating the overlap of genes with significantly enriched H3K9ac peaks in leaves of WT plants at 24 h after herbivore damage (HD) compared to undamaged (UD) WT plants, *hac1* and HDA6‐OX2 plants with HD, as determined by chromatin immunoprecipitation (ChIP)‐sequencing analysis. (b) Gene ontology (GO) analysis of 166 genes in the categories (WT_HD > WT_UD; WT_HD > HDA6‐OX2_HD; and WT_HD > *hac1*_HD). The top 20 GO categories are shown. (c) Positional profiles of H3K9ac on *PDF1.2* and *ERF13* in leaves of WT, *hac1* and HDA6‐OX2 plants. The normalized coverage (averaged read counts per 25 bp window) for *PDF1.2* and *ERF13* are shown in the respective upper panels. The position of the coding DNA sequences, the untranslated regions and the regions amplified by polymerase chain reaction (PCR) are shown at the bottom. (d) ChIP analysis combined with quantitative PCR to determine H3K9ac levels in defence‐related genes (*PDF1.2* and *ERF13*) in leaves of UD and HD WT, *hac1* and HDA6‐OX2 plants. Individual data points are shown with means and standard errors (*n* = 3). An asterisk(s) indicates significant differences compared to damaged WT, as determined by ANOVA with Holm's sequential Bonferroni post hoc test (**p < 0.01; *0.01 ≤ *p* < 0.05). ns, not significant (*p* ≥ 0.05).

We performed ChIP‐Seq to identify genes with enriched histone acetylation in Arabidopsis leaves in response to *S. litura* attack (Supporting Information S1: Figure S2). Since histone H3 tail acetylation tends to have a more destabilizing effect on nucleosome structure than H4 tail acetylation (Gansen et al. 2015), we focused our study on histone H3 tail acetylation. Our analyses using anti‐acetyl histone H3K9 antibody revealed that H3K9ac was up‐ and downregulated at 576 genes and 2837 genes, respectively, in leaves exposed to *S. litura* for 24 h (Figure 1a, Supporting Information S1: Table S2). Among these upregulated genes, 495 genes showed lower H3K9ac enrichment in *hac1*, a T‐DNA insertion mutant plant deficient in HAC1, which is known to be involved in plant defence responses (Fina et al. 2017; Onosato et al. 2022; Singh et al. 2014; Zheng et al. 2019). Similarly, the lower enrichment in 176 genes was observed in HDA6‐OX2, transgenic plants overexpressing *HDA6* (Supporting Information S1: Figure S3), an HDAC involved in the deacetylation of lysine residues in histone H3 and H4 N‐tails, excluding H4K16 (To et al. 2011) (Figure 1a, Supporting Information S1: Table S3).

A total of 166 genes showed enriched H3K9ac in leaves of infested WT plants but not infested *hac1* or HDA6‐OX2 plants (Figure 1a). GO analysis of these genes showed significant enrichment of GO terms related to stress response, including terms involved in ‘response to wounding’ (Figure 1b, Supporting Information S1: Table S4). Notably, of H3K9ac levels of the 166 genes, herbivore‐responsive genes such as *PDF1.2*, ethylene response factor 13 (*ERF13*) and PBS1‐like 27 (*PBL27*) appeared likely to be the ones whose H3K9ac status is most substantially altered by HAC1 and HDA6 in response to *S. litura* attack (Supporting Information S1: Tables S5 and S6). However, since the acetylation status of *PBL27* was not confirmed by ChIP analysis combined with qPCR (ChIP‐qPCR) (Supporting Information S1: Figure S4), this gene was not included in the subsequent analyses of this study. The two genes, *PDF1.2* and *ERF13*, which were finally selected as the target genes of our study have been shown to play important roles in the defence response of Arabidopsis against *S. litura* larvae (Arimura 2021; Desaki et al. 2023; Miyamoto et al. 2019; Uemura et al. 2020).

Importantly, the H3K9ac patterns on these genes showed remarkable differences: the H3K9ac level peaked primarily at the 5’ flanking (promoter) region of *PDF1.2*, while it instead peaked within the gene body of most genes, including *ERF13* (Figure 1c). Further, ChIP‐qPCR confirmed the increased level of H3K9ac at the promoter region of *PDF1.2* and within the *ERF13* gene body in leaves of WT plants but not in leaves of *hac1* or HDA6‐OX2 plants (Figure 1d). Furthermore, since *hda6*, a T‐DNA insertion mutant plant deficient in HDA6, showed enriched H3K9ac in undamaged leaves compared to undamaged WT leaves, HDA6 was confirmed to be responsible for H3K9ac at the corresponding regions of *PDF1.2* and *ERF13* (Supporting Information S1: Figure S5a).

Notably, the *PDF1.2* gene body and the promoter region of *ERF13* were not enriched in acetylation levels in the infested WT leaves (Supporting Information S1: Figure S6a and S6b), indicating that H3K9ac occurs specifically at the promoter region of *PDF1.2* and the gene body of *ERF13*. Therefore, our focus in the current study was on the acetylation status specifically at the *PDF1.2* promoter region and the *ERF13* gene body.

### 3.2 Early Response of Histone Acetylation in Leaves Attacked by *S. litura*

**Figure 2:**
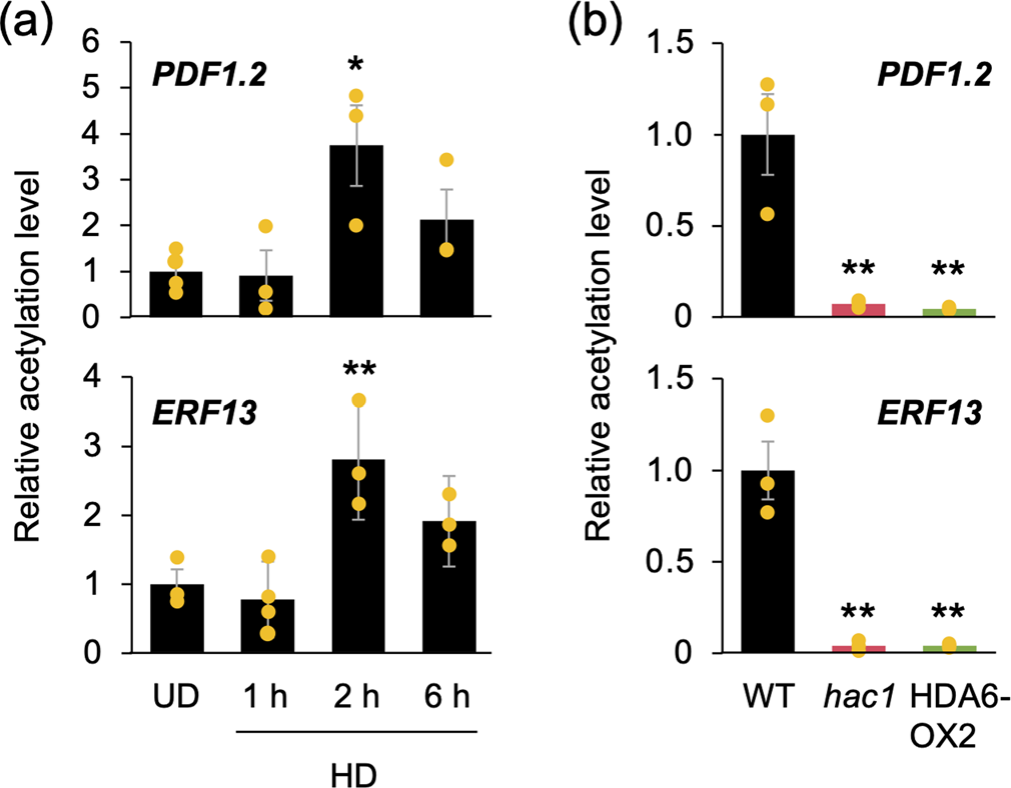
Early response of H3K9 acetylation (H3K9ac) in leaves damaged by *Spodoptera litura*. (a) Changes in H3K9ac levels at *PDF1.2* and *ERF13* in leaves of undamaged (UD) wild‐type (WT) plants and those in response to damage by *S. litura* larvae (HD) from 1 to 6 h. (b) H3K9ac levels at both genes in leaves of WT, *hac1* mutants and HDA6‐overexpressing (HDA6‐OX2) plants in response to HD for 2 h. Individual data points are shown with means and standard errors (*n* = 3−4). An asterisk(s) indicates significant differences compared to UD (a) or WT (b), as determined by ANOVA with Holm's sequential Bonferroni post hoc test (**p < 0.01; *0.01 ≤ *p* < 0.05). [Color figure can be viewed at wileyonlinelibrary.com]

We examined the time course of H3K9ac levels in leaves after *S. litura* damage. Our results showed that in WT plants, the enriched H3K9ac at *PDF1.2* and *ERF13* started as early as 2 h after damage (Figure 2a). Since no enriched H3K9ac at *PDF1.2* or *ERF13* was observed in the whole damaged leaf at 1 h after *S. litura* damage, we then collected only locally damaged areas and analysed their H3K9ac levels. As a result, no significant enriched histone H3K9ac was found at either *PDF1.2* or *ERF13*, indicating that histone acetylation after *S. litura* damage is enriched at these genes at least after 2 h, regardless of the location within the damaged leaf (Supporting Information S1: Figure S7).

**Figure 3:**
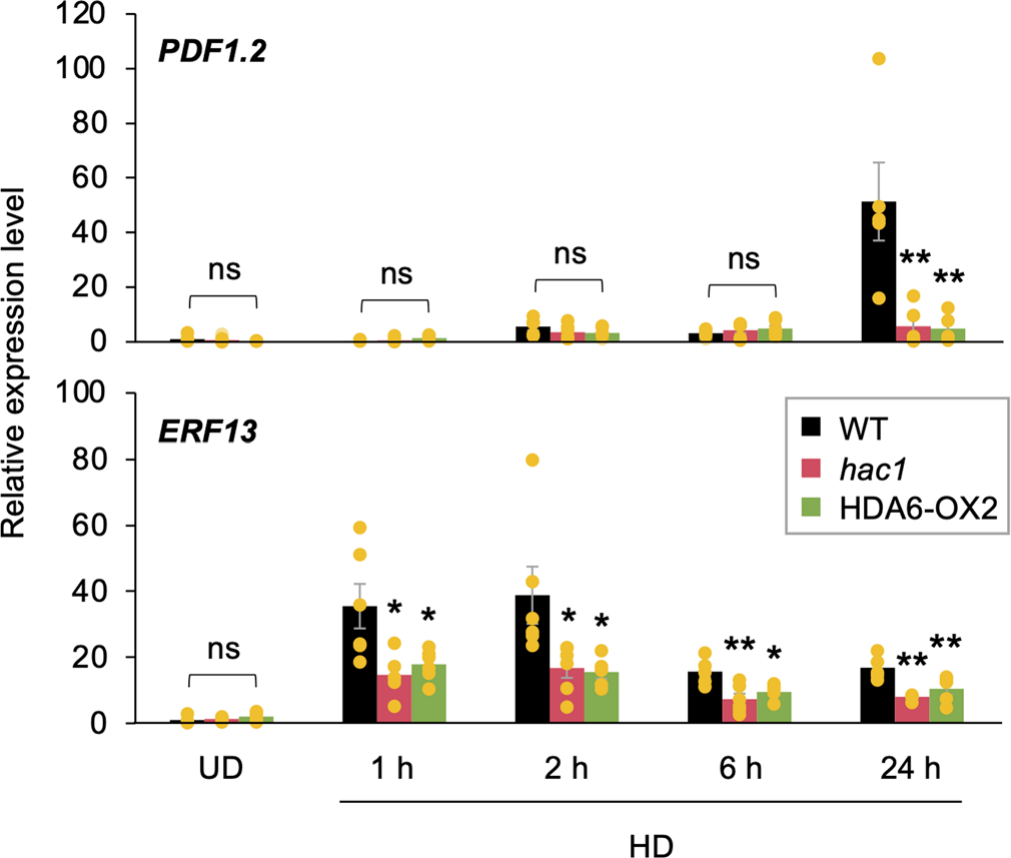
Temporal changes in defence‐related gene expression in leaves of undamaged (UD) wild‐type (WT), *hac1* mutant and HDA6‐overexpressing (HDA6‐OX2) plants and those in response to damage by *Spodoptera litura* larvae (HD) from 1 to 24 h. Individual data points are presented with means and standard errors (*n* = 5–6). An asterisk(s) indicates significant differences compared to WT at the corresponding time point after damage, as determined by ANOVA with Holm's sequential Bonferroni post hoc test (**p < 0.01; *0.01 ≤ *p* < 0.05). ns, not significant (*p* ≥ 0.05). [Color figure can be viewed at wileyonlinelibrary.com]

The level of H3K9ac at both genes was significantly lower in leaves of *hac1* and HDA6‐OX2 plants than in WT leaves at 2 h (Figure 2b). Such histone acetylation affected the expression levels of the corresponding genes in the early stages of herbivore damage but with a time lag. This was typically seen in the delayed induction of *PDF1.2* expression observed 24 h after the onset of herbivore damage (Figure 3). More interestingly, even before H3K9ac reached sufficient levels at 2 h, *ERF13* expression was already stimulated at 1 h after the onset of the damage. However, in contrast to *PDF1.2*, whose expression was strikingly deficient in *hac1* and HDA6‐OX2 leaves (89% and 91% lower than that in WT leaves, respectively, after 24 h), *ERF13* expression was 59% and 50% lower, respectively, in *hac1* and HDA6‐OX2 leaves after 1 h. These results indicate that H3K9ac is only partly responsible for the transcriptional regulation of *ERF13*. In contrast, *hda6* plants showed an increase in expression levels of both *PDF1.2* and *ERF13* in undamaged leaves compared to undamaged WT leaves (Supporting Information S1: Figure S5b).

Intriguingly, the regulations of histone acetylation/deacetylation at *PDF1.2* and *ERF13* were found to be independent of typical herbivore damage signals, including wound stimuli and OS factors that induce expression of *PDF1.2* and *ERF13* (Desaki et al. 2023), as H3K9ac at both of these genes was unaffected at 1 and 2 h after MD or treatment with OS isolated from *S. litura* larvae (Supporting Information S1: Figure S8).

### 3.3 HDA6 Interacts With TPL/TPR Corepressors

**Figure 4:**
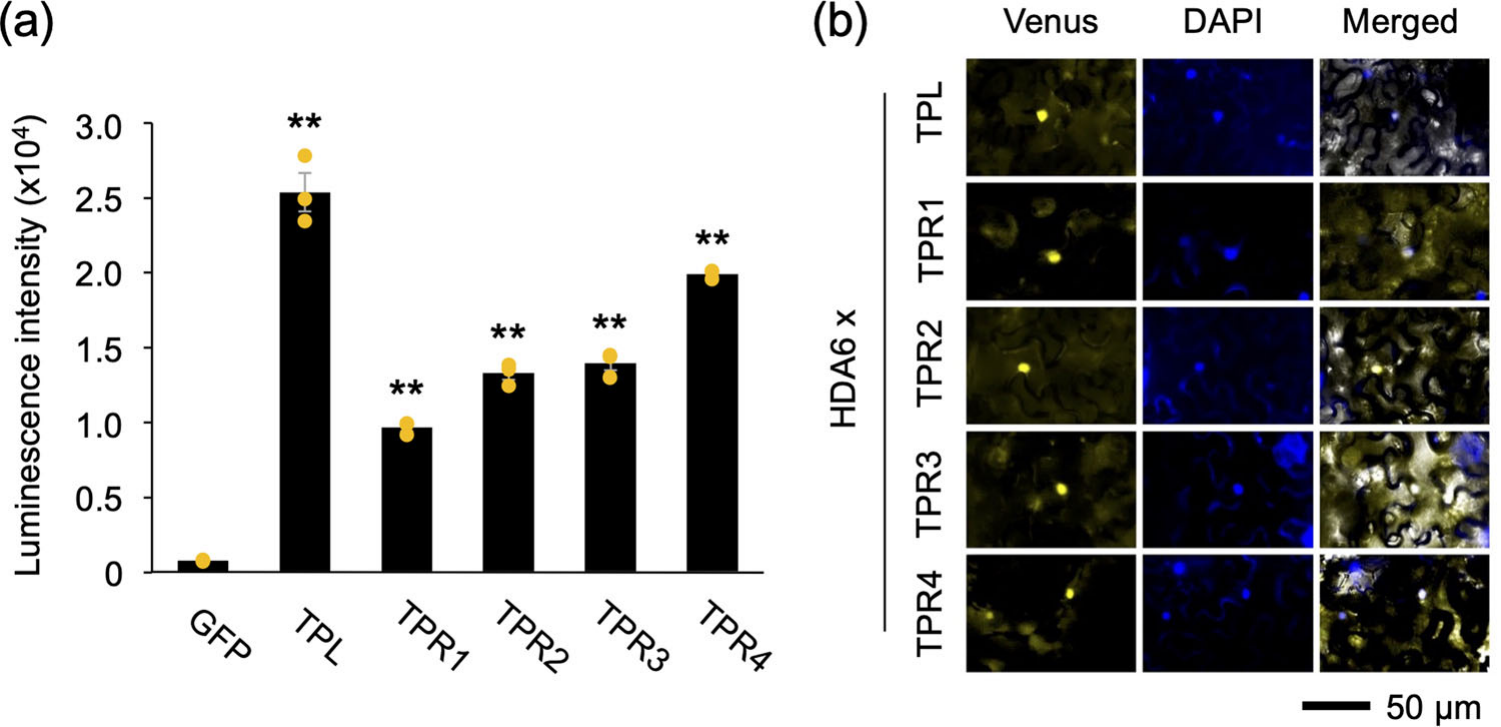
Interaction between HDA6 and TPL/TPR proteins. (a) Quantification of the interaction intensity between biotinylated HDA6 protein and FLAG‐conjugated proteins (including TPL/TPR proteins and GFP as control) using the AlphaScreen system. Individual data points are presented with mean values and standard errors (*n* = 3). Asterisks indicate significant differences compared to the GFP control, as determined by ANOVA with Holm's sequential Bonferroni post‐hoc test (**p < 0.01). (b) Visualization of the in planta interaction between HDA6 fused to the N‐terminal fragment of Venus and each TPL/TPR protein fused to the C‐terminal fragment of Venus in *Nicotiana benthamiana* leaf cells, using bimolecular fluorescence complementation analysis. The images show the reconstructed Venus signal, DAPI (4′,6‐diamidino‐2‐phenylindole) fluorescence and the merged image with a bright field. [Color figure can be viewed at wileyonlinelibrary.com]

HDACs, such as HDA6, lack the ability to bind directly to DNA, suggesting that they are essentially recruited to their target loci by an additional factor(s) (Jiang et al. 2020). In this light, HDA6 has been predicted to be recruited by TPL to deacetylate histones and act as a repressor of gene expression in Arabidopsis (Saini and Nandi 2022; Wang, Kim, and Somers 2013). We hypothesized that upon biotic stress, the HDA6/TPL corepressor complex could be released, leading to histone acetylation of previously repressed chromatin regions and subsequent transcriptional activation. To test the validity of this theoretical model of plant−herbivore interactions, we performed a screening for TPL/TPR corepressors that interact with HDA6. While interaction between TPL and HDA6 has been demonstrated (Wang, Kim, and Somers 2013), actual interaction between other TPRs and HDA6 has not been confirmed. Hence, we evaluated the in vitro interaction between the biotinylated recombinant HDA6 protein and FLAG‐conjugated TPL/TPR, using the AlphaScreen system. The resulting luminescence intensities showed that the HDA6 protein interacted strongly with TPL and all TPRs tested compared to the GFP control (Figure 4a). Furthermore, all these interactions were confirmed *in planta* using BiFC assays. Concomitant expression of HDA6 fused to the N‐terminal fragment of Venus and TPL/TPR fused to the C‐terminal fragment of Venus in *N. benthamiana* leaf cells resulted in fluorescent signals of the reconstructed Venus proteins in the nuclei (Figure 4b).

### 3.4 Involvement of TPL/TPR Corepressors in Antiherbivore Response

Next, we generated transgenic plants overexpressing TPL/TPR, which showed interaction with HDA6, to evaluate their effect on H3K9ac at *PDF1.2* and *ERF13* and on their expression. The expression levels of the corresponding genes in each homozygous line were analysed and the results showed that the expression levels of the *TPL*, *TPR1*, *TPR2*, *TPR3* and *TPR4* genes were most significantly increased in TPL‐OX2, TPR1‐OX3, TPR2‐OX3, TPR3‐OX3 and TPR4‐OX2 plants, respectively, compared with those in WT plants (Supporting Information S1: Figure S3). Therefore, we focused on using these T3 lines for subsequent analyses.

**Figure 5:**
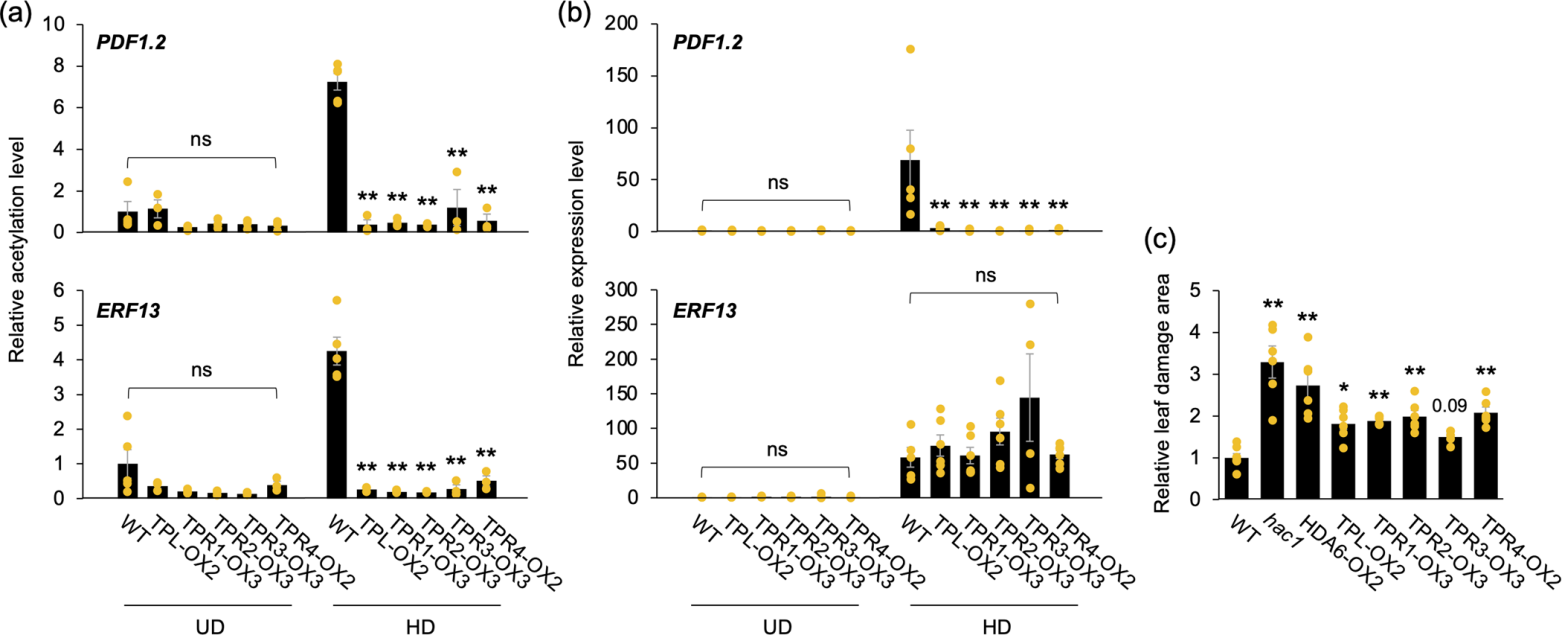
TPL/TPRs involved in histone deacetylation and defence properties. (a) H3K9 acetylation (H3K9ac) levels at *PDF1.2* and *ERF13* in undamaged (UD) wild‐type (WT) and TPL/TPR overexpressed plants (TPL/TPR‐OX) and those in response to damage by *Spodoptera litura* larvae (HD) for 2 h. (b) The expression levels of *PDF1.2* and *ERF13* in leaves of undamaged WT and TPL/TPR‐OX plants and those in response to HD for 24 h. (c) Relative leaf damage area of WT, TPL/TPR‐OX, *hac1* and HDA6‐OX2 plants damaged by *S. litura* larvae for 24 h. Individual data points are presented with mean values and standard errors (*n* = 3–5, *n* = 4–6 and *n* = 5–6 for a, b and c, respectively). An asterisk(s) indicates significant differences compared to WT, as determined by ANOVA with Holm's sequential Bonferroni post hoc test (**p < 0.01; *0.01 ≤ *p* < 0.05). Data shown with a *p* value are marginally different from WT. ns, not significant (*p* ≥ 0.05). [Color figure can be viewed at wileyonlinelibrary.com]

In response to damage by *S. litura* larvae for 2 h, transgenic plants overexpressing the respective TPL and TPRs showed significantly lower levels of histone acetylation at *PDF1.2* and *ERF13* in their leaves than WT plants (Figure 5a). The same was true for the expression level of *PDF1.2* in 24 h damaged leaves, but no difference was observed in the expression level of *ERF13* between leaves of WT and transgenic plants (Figure 5b).

Similar to *hac1* and HDA6‐OX2, all transgenic plants overexpressing TPL/TPRs exhibited more leaf feeding damage by *S. litura* larvae compared to WT plants (note that TPR3‐OX3 plants were only marginally different; Figure 5c). These results suggest that all TPL/TPRs act as repressors of antiherbivore responses.

### 3.5 TPL Is Required to Recruit HDA6 to the *PDF1.2* Promoter Region

**Figure 6:**
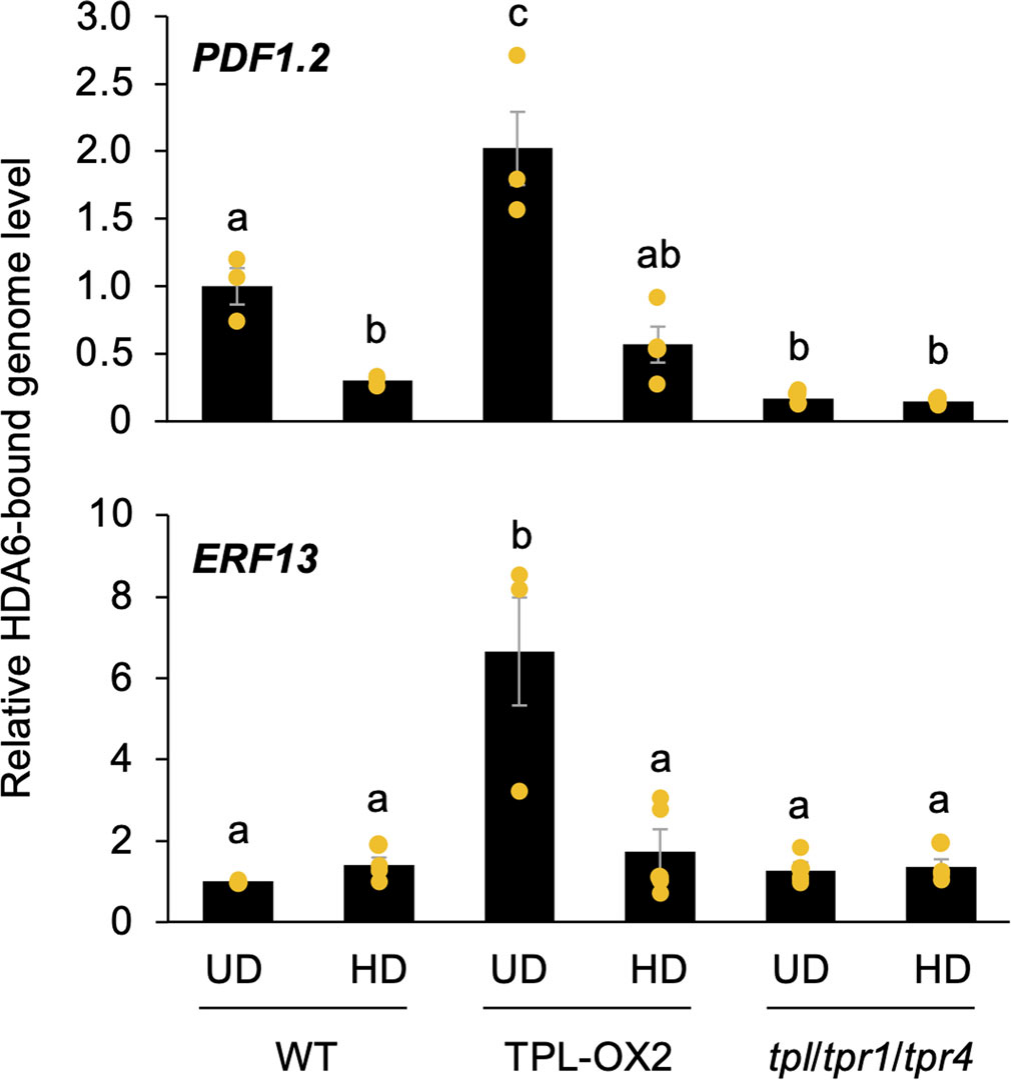
HDA6 recruitment to the *PDF1.2* promoter in a TPL‐dependent manner. Immunoprecipitation of proteins in leaves of undamaged (UD) wild‐type (WT), TPL‐OX2 and *tpl*/*tpr1*/*tpr4* plants and those damaged by *Spodoptera litura* larvae (HD) for 2 h was performed using the anti‐HDA6 antibody. The *PDF1.2* promoter and the *ERF13* gene body bound by the immunoprecipitated HDA6 proteins were quantified. Individual data points are presented with mean values and standard errors (*n* = 3–5). Means indicated by different small letters are significantly different based on ANOVA with post hoc Tukey HSD (*p* < 0.05). [Color figure can be viewed at wileyonlinelibrary.com]

To assess the possibility that TPL is a scaffold protein that recruits HDA6 to the promoter region of *PDF1.2* and the gene body of *ERF13*, we performed ChIP‐qPCR for *PDF1.2* and *ERF13*‐bound proteins in leaves of undamaged and damaged WT, TPL‐OX2 and *tpl*/*tpr1*/*tpr4* (the triple mutant of *TPL*, *TPR1* and *TPR4*) plants using the anti‐HDA6 antibody. The results showed that the levels of the HDA6‐bound *PDF1.2* promoter region were lower in leaves of WT plants damaged with *S. litura* larvae for 2 h, compared to those in undamaged WT leaves (Figure 6). A similar trend was observed in *tpl*/*tpr1*/*tpr4* leaves, both in undamaged and damaged plants. However, the levels of the HDA6‐bound *PDF1.2* promoter region were higher in undamaged TPL‐OX2 leaves, but not in damaged TPL‐OX2 leaves, compared to undamaged WT leaves. On the other hand, the levels of HDA6‐bound *ERF13* were enriched only in leaves of TPL‐OX2 plants. However, it was not reduced in leaves of WT plants damaged by *S. litura* larvae, or in *tpl*/*tpr1*/*tpr4* plants with or without *S. litura* damage, compared to undamaged WT plants. All these results were confirmed with the other qPCR primer sets, although one set of primers showed a less significant interaction of HDA6 at the *ERF13* gene body (Supporting Information S1: Figures S6a and S6c). Taken together, these results suggest that HDA6 targets chromatin at the *PDF1.2* promoter region and possibly the *ERF13* gene body in a TPL‐dependent manner in undamaged plants.

### 3.6 TPL Functions Negatively in ERF13‐Mediated *PDF1.2* Regulation

**Figure 7:**
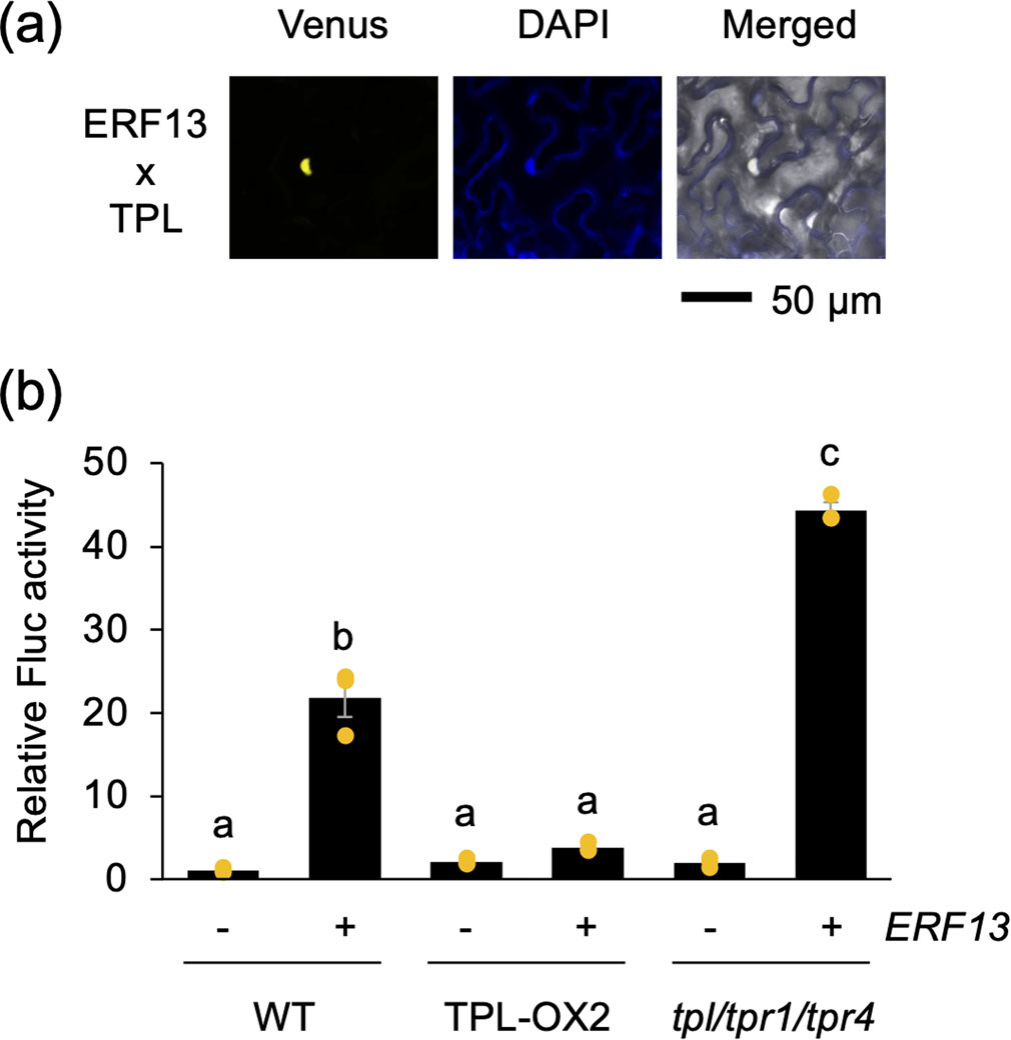
TPL acts as repressor of ERF13‐mediated *PDF1.2* transcription. (a) Visualization of the in planta interaction between ERF13 fused to the N‐terminal fragment of Venus and TPL protein fused to the C‐terminal fragment of Venus in *Nicotiana benthamiana* leaf cells, using bimolecular fluorescence complementation analysis. The images show the reconstructed Venus signal, DAPI (4′,6‐diamidino‐2‐phenylindole) fluorescence and the merged image with a bright field. (b) Transient activation of firefly luciferase (Fluc) reporter gene under the control of *PDF1.2* promoter following expression with (+) or without (−) *ERF13* in protoplasts prepared from Arabidopsis WT, TPL‐OX2 and *tpl*/*tpr1/tpr4* leaves. Individual data points are presented with mean values and standard errors (*n* = 3). Means indicated by different small letters are significantly different based on ANOVA with post hoc Tukey HSD (*p* < 0.05). [Color figure can be viewed at wileyonlinelibrary.com]

TPL has been shown to interact with ERFs in vitro (Causier et al. 2012). Concomitant expression of ERF13 fused to the N‐terminal fragment of Venus and TPL fused to the C‐terminal fragment of Venus in *N. benthamiana* leaf cells resulted in fluorescent signals of the reconstructed Venus proteins in the nuclei (Figure 7a), confirming their in planta interactions. The same was true for the interactions between ERF13 and TPR3 or TPR4, but not TPR1 or TPR2, fused to the N‐ and C‐terminal fragments of Venus, respectively (Supporting Information S1: Figure S9).

Based on these resutls, and to further assess the significance of the interactions between ERF13 and TPL/TPRs, ERF13 was coexpressed with a Fluc reporter gene under the control of the *PDF1.2* promoter region (PDF1.2P) in protoplasts prepared from WT, TPL‐OX2 and *tpl*/*tpr1*/*tpr4* leaves. PDF1.2P contains the GCC box (ERF‐binding *cis*‐element [Fujimoto et al. 2000]) located at –255 to –261, which promotes *S. litura*‐inducible transcription of the gene (Miyamoto et al. 2019). The data showed that when PDF1.2P::Fluc and ERF13 were co‐expressed in protoplasts, an increased level of Fluc activity was observed in WT protoplasts, while it was lower in TPL‐OX2 protoplasts and higher in *tpl*/*tpr1*/*tpr4* protoplasts (Figure 7b), indicating that TPL has a negative function in ERF13‐mediated *PDF1.2* regulation.

## 4 Discussion

**Figure 8:**
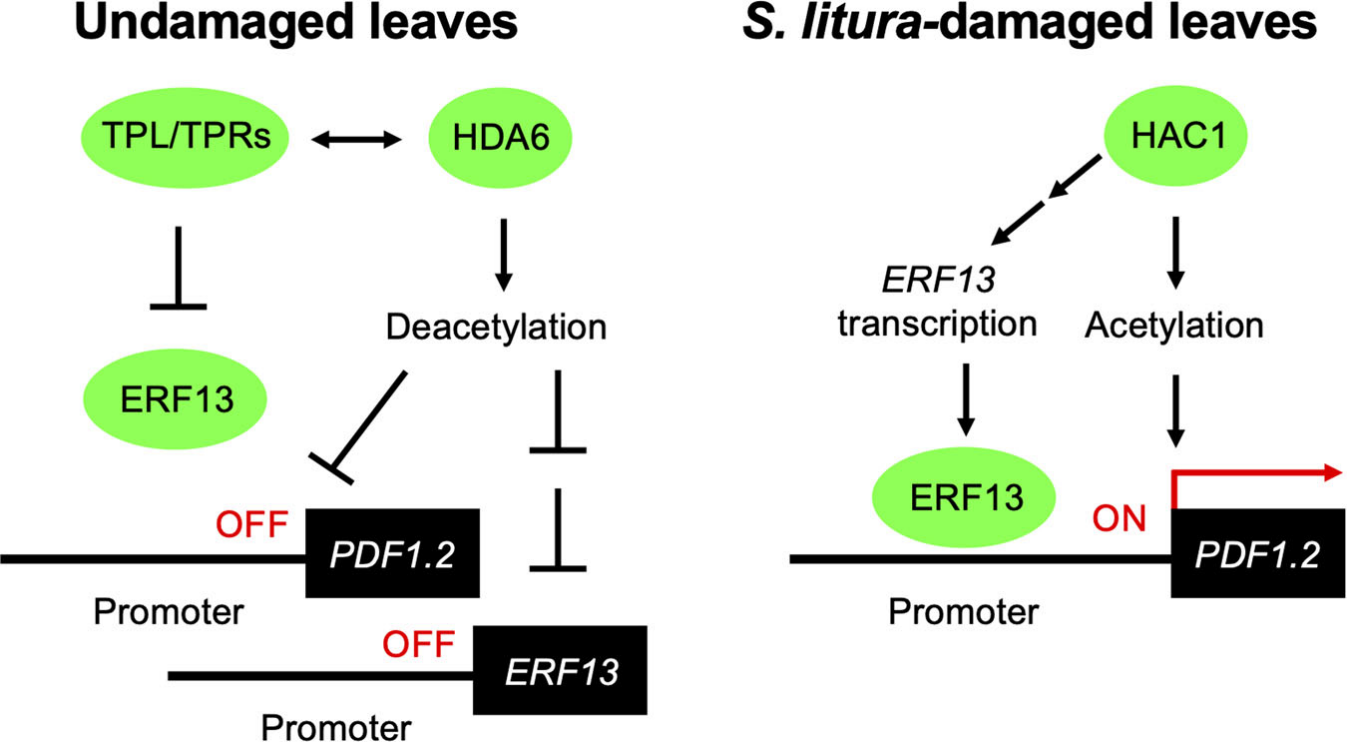
A possible model for the switching mode of *PDF1.2* transcription in Arabidopsis leaves between the undamaged, steady state and the *Spodoptera litura*‐damaged state. This switch occurs within 2 h after the onset of the herbivore damage. TPL/TPRs are often scaffolded on the nucleosome (Ma et al. 2017) and then interact with HDA6 and/or ERF13 in undamaged leaves. During herbivory, histone acetyltransferases, including HAC1, selectively acetylate chromatin by binding to DNA‐binding proteins (Chen, Guo, and Dong 2024) on the *PDF1.2* promoter and the *ERF13* gene body, at least within 2 h of damage onset. The arrows and bars indicate positive and negative interactions, respectively. Double arrows and bars indicate possible indirect interactions. [Color figure can be viewed at wileyonlinelibrary.com]

We propose a model for the negative regulation of the expression of the defence gene *PDF1.2* by the HDA6‐TPL/TPR system in healthy Arabidopsis plants (Figure 8). However, damage by *S. litura* larvae causes rapid H3K9ac at the 5’ flanking promoter region of *PDF1.2*, leading to transcriptional activation within at least 2 h after the onset of the herbivore attack. In the promoter region, several transcription factors form a complex with the jasmonate ZIM domain (JAZ) repressor and TPL/TPRs (e.g., TPR2) that interact with JAZ through the ERF‐associated amphiphilic repression (EAR) domain either directly or indirectly via NOVEL INTERACTOR OF JAZ (NINJA) and EAR motif‐containing adaptor proteins (ECAP) (Li et al. 2020; Pauwels et al. 2010). TPL/TPRs can also interact with transcription factors containing repression domains (RDs) with conserved amino acid sequences, such as DLNxxP, R/KLFGV, TLxLF, ERF‐associated EAR domain with LxLxL amino acid sequences or less characterized motifs, resulting in the transcriptional repression (Causier et al. 2012). This regulatory mechanism ensures the effectiveness of the negative mode of *PDF1.2* expression in Arabidopsis, which is negatively regulated by TPL, and possibly TPRs 3 and 4 (Figure 7 and Supporting Information S1: Figure S9).

In addition, our results (Figure 5), together with a previous report (Wang, Kim, and Somers 2013), show that all of the TPL/TPRs play a critical role in facilitating H3K9 deacetylation through their interaction with HDA6, leading to dramatically enhanced transcriptional repression of *PDF1.2*. All TPL/TPRs that bind to HDA6 were found to play almost the same role in regulating H3K9ac and gene expression in the *PDF1.2* promoter region, causing a negative regulation of plant defence against herbivores (Figure 5). TPL/TPRs are known for their tetramerization (Ke et al. 2015; Martin‐Arevalillo et al. 2017), in which TPL/TPRs can interact with each other through its TOPLESS domain (TPD), allowing their oligomerization and TPD‐nucleosome interaction (Ma et al. 2017). Such redundant function of TPL/TPRs has been shown in rice, in which TPL/TPR1/TPR2 work in concert with OsbHLH061/OsPRI1 to negatively control long‐distance iron transport in the plants (Wang et al. 2022).

During herbivory, the HDA6‐TPL/TPR complex is likely to disengage from the *PDF1.2* promoter in infested leaf cells (Figure 6), causing some of the TPL/TPRs (e.g., TPR1, and possibly TPL and TPR4) to become SUMOylated and inactive (Niu et al. 2019). This probably allows HAC1, and possibly other members of the HAT family, to access chromatin. As described above, HAC1, HAC5 and HAM1, play a critical role in regulating the histone H3 and H4 acetylation during the β‐ocimene response in Arabidopsis (Onosato et al. 2022). Similarly, HAC1 and HAC5 act in the transcriptional regulation of *pathogenesis‐related gene 1* (*PR1*) by forming a complex with a salicylic acid‐dependent activator known as a non‐expressor of PR1 (NPR1) (Jin et al. 2018). Thus, several HATs may be involved in plant defence responses.

It should also be noted that the HDA6‐TPL/TPR complex can be continuously maintained on the *ERF13* gene body in Arabidopsis plants even after *S. litura* attack. Considering that TPL/TPRs certainly have great potential to contribute to the recruitment of HDA6 (Figure 6) and that *hda6* plants show defective deacetylation of *PDF1.2* and *ERF13* in healthy leaves (Supporting Information S1: Figure S5), we propose the hypothesis that basal levels of TPL/TPRs are not sufficient to control HDA6 recruitment and/or that some fractions of HDA6 function at the *ERF13* gene body in a manner independent of TPL/TPRs.

More interestingly, although H3K9 is highly acetylated at the *ERF13* gene body in leaves in response to *S. litura* attack, the influence of H3K9ac on *ERF13* expression appears to be less pronounced than that on the expression of *PDF1.2*, whose promoter region is predominantly regulated by H3K9ac (Figures 1, 2 and 3). These findings would be consistent with *ERF13* being targeted by H3K9ac but not being transcriptionally repressed by TPL/TPRs (Figure 5), suggesting that H3K9ac of the proximal chromatin region and the scaffolding of TPL/TPRs at the gene body may not always allow dominant regulation of the transcription. We predict that HAC1 and HDA6 are extensively involved not only in regulating H3K9ac at the *ERF13* gene body, but also in upstream signalling for *ERF13* transcription, as a large number of genes are potentially regulated by both HAC1 and HDA6 in Arabidopsis leaves during *S. litura* attack (Figure 1a). This would be consistent with the finding that HAC1/HDA6‐dependent expression of *ERF13* was upregulated at 1 h after the onset of herbivore attack, when H3K9 levels at *ERF13* were still not yet elevated (Figure 3), suggesting that *ERF13* transcription occurs independently of elevated H3K9 levels at its gene body. In addition, since *ERF13* expression was not suppressed in any of the TPL/TPR‐OX plants (Figure 5b), the HAC1/HDA6‐dependent upstream factor(s) are likely to be regulated independently of TPL/TPRs.

The polysaccharide elicitor in the OS of *S. litura* larvae has been shown to induce ethylene production and subsequent upregulation of *PDF1.2* expressions (Uemura et al. 2020). The signalling cascade is mediated by the cytoplasmic kinase PBL27 and PBL27‐dependent transcription of *ERF13*, leading to activation of *PDF1.2* expression (Desaki et al. 2023). However, since H3K9ac levels at *PDF1.2* and *ERF13* are not increased by application of OS (Supporting Information S1: Figure S8), the histone acetylation mechanism is likely to be independent of both single wound stimuli and oral factors. However, herbivore damage signals also involve other, multiple factors such as continuous leaf damage (Arimura et al. 2008; Bricchi et al. 2010). In addition, TPL has been shown to react with caryophyllene, a herbivore‐induced plant volatile, to induce the expression of stress‐responsive genes in tobacco (Nagashima et al. 2019), suggesting that such volatile infochemicals may be master regulators of histone acetylation. However, it remains to be clarified whether these terpenoids are synthesized de novo within 2 h after the onset of herbivore damage (Maffei, Mithöfer, and Boland 2007). Such diverse herbivore damage signals, in addition to oral factors resulting from the complex performance of the herbivore during feeding, may contribute to the early response of histone acetylation in plants.

In conclusion, we address the *PDF1.2* transcriptional mode switched by feedback regulation, mediated by the histone acetylation system consisting of HAC1, HDA6 and TPL/TPRs, between undamaged and *S. litura*‐damaged leaf status. Further analysis using more genetic tools would help to understand our model in more detail.

## Conflicts of Interest

The authors declare no conflicts of interest.

## Supporting information

Supporting information.

Supporting information.

## Data Availability

The data that support the findings of this study are available from the corresponding author upon reasonable request.
