# Supplementary material for: Histone Modification‐Dependent Transcriptional Regulation of Defence Genes in Early Response of Arabidopsis to Spodoptera litura Attack
Source: Plant Cell Environ. 2024 Dec 25;48(5):3257–68. doi: 10.1111/pce.15345 (PMC11963488; doi:10.1111/pce.15345)
Supplement: Supplementary file 1 — Supporting information. [file PCE-48-3257-s001.pdf]

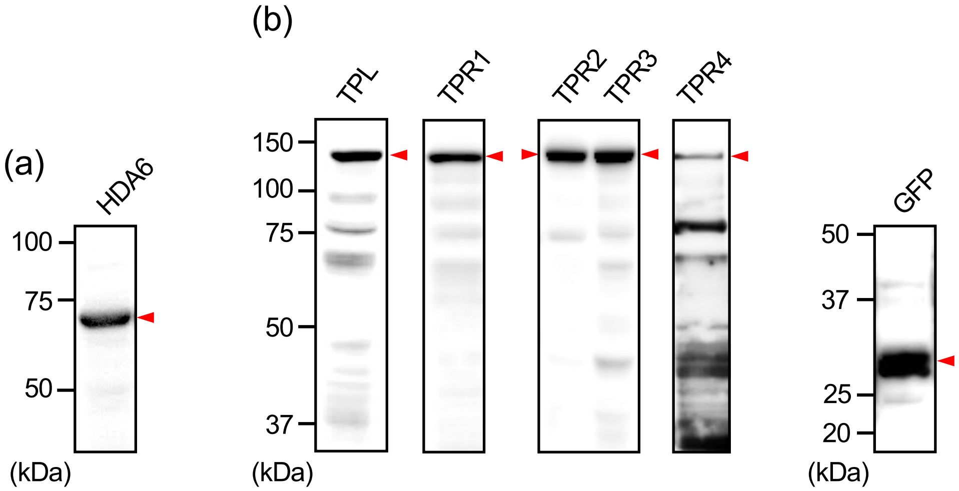

(a)
HDA6
100
75
50
(kDa)
(b)
TPL
TPR1
TPR2
TPR3
TPR4
150
100
75
50
37
(kDa)
GFP
50
37
25
20
(kDa)

Figure S1

(a)

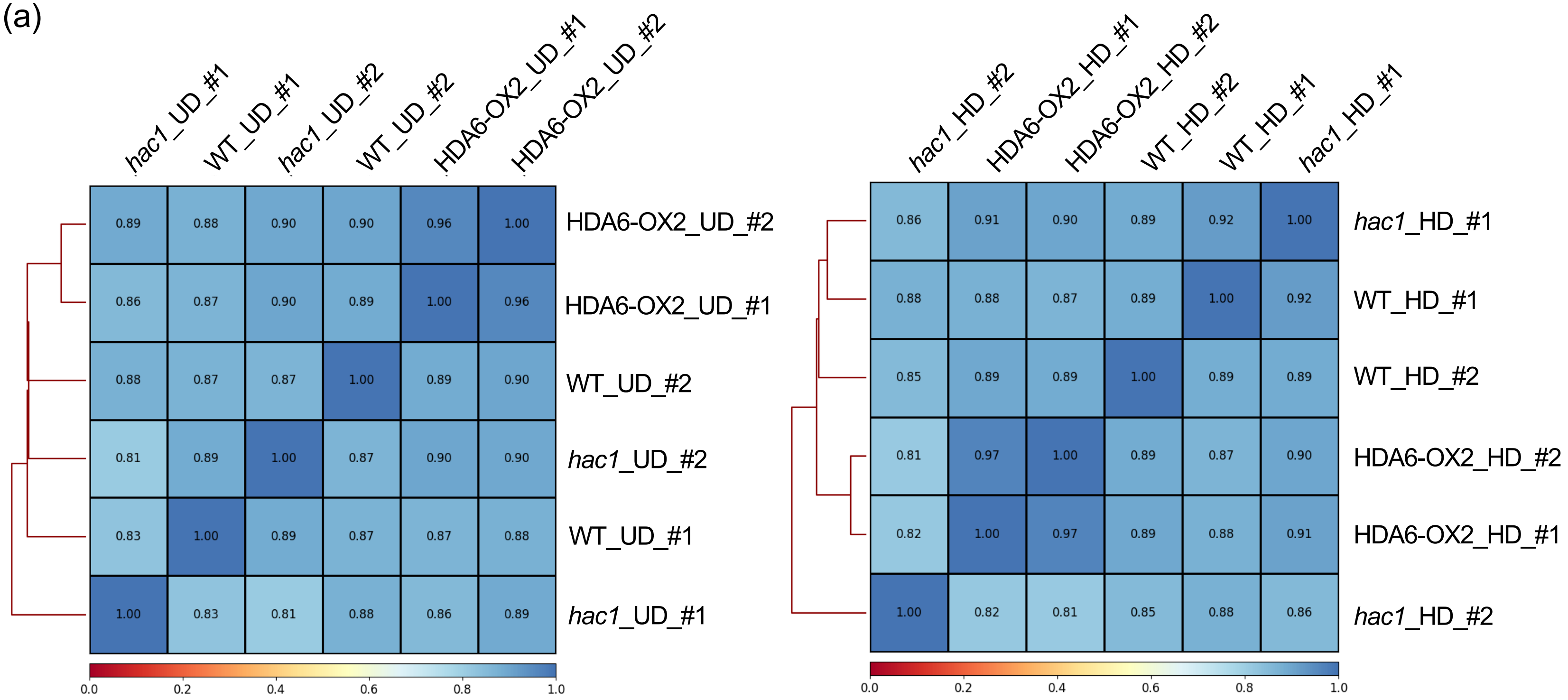
hac1_UD_#1
WT_UD_#1
hac1_UD_#2
WT_UD_#2
HDA6-OX2_UD_#1
HDA6-OX2_UD_#2
0.89 0.88 0.90 0.90 0.96 1.00 HDA6-OX2_UD_#2
0.86 0.87 0.90 0.89 1.00 0.96 HDA6-OX2_UD_#1
0.88 0.87 0.87 1.00 0.89 0.90 WT_UD_#2
0.81 0.89 1.00 0.87 0.90 0.90 hac1_UD_#2
0.83 1.00 0.89 0.87 0.87 0.88 WT_UD_#1
1.00 0.83 0.81 0.88 0.86 0.89 hac1_UD_#1
0.0
0.2
0.4
0.6
0.8
1.0
hac1_HD_#2
HDA6-OX2_HD_#1
HDA6-OX2_HD_#2
WT_HD_#2
WT_HD_#1
hac1_HD_#1
0.86 0.91 0.90 0.89 0.92 1.00 hac1_HD_#1
0.88 0.88 0.87 0.89 1.00 0.92 WT_HD_#1
0.85 0.89 0.89 1.00 0.89 0.89 WT_HD_#2
0.81 0.97 1.00 0.89 0.87 0.90 HDA6-OX2_HD_#2
0.82 1.00 0.97 0.89 0.88 0.91 HDA6-OX2_HD_#1
1.00 0.82 0.81 0.85 0.88 0.86 hac1_HD_#2
0.0
0.2
0.4
0.6
0.8
1.0

Figure S2

(b)

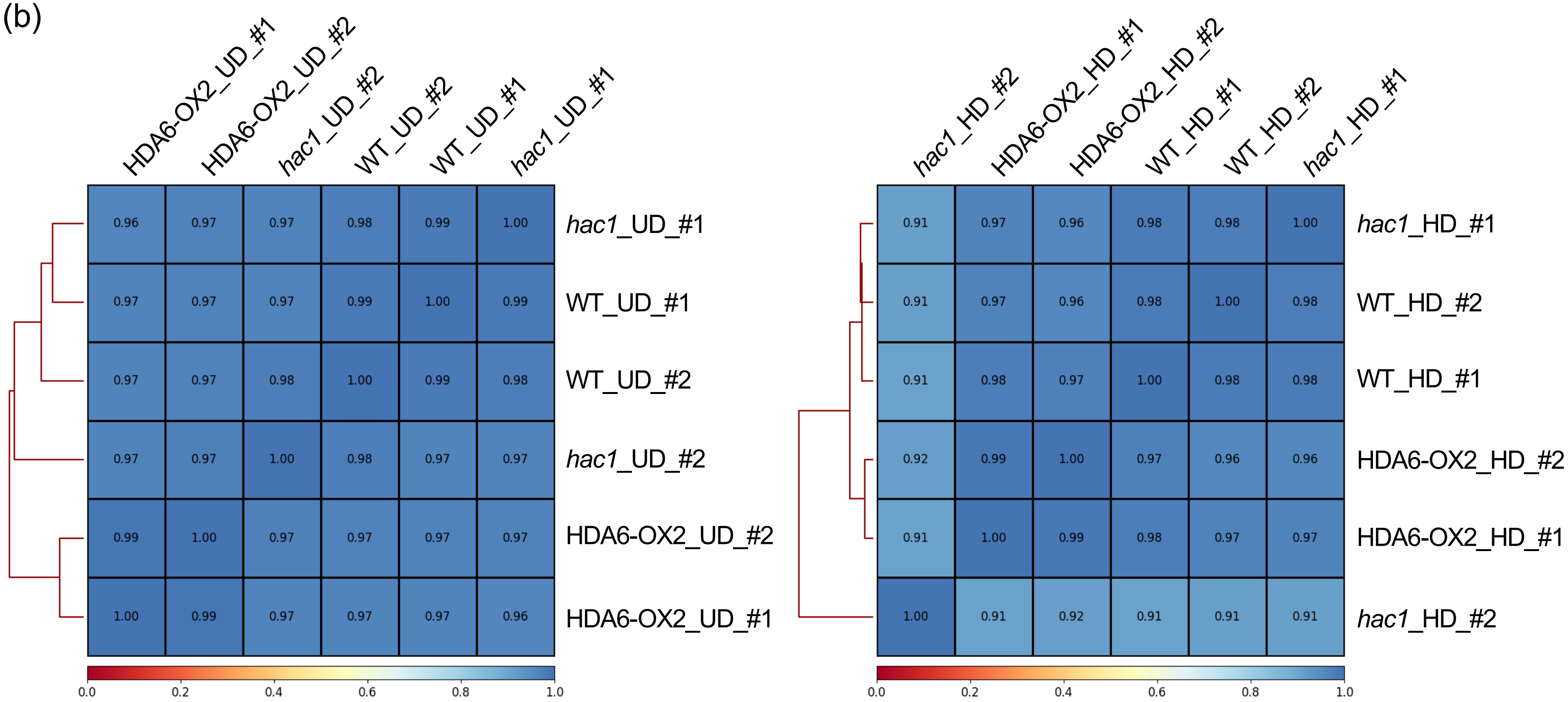

HDA6-OX2_UD_#1
HDA6-OX2_UD_#2
hac1_UD_#2
WT_UD_#2
WT_UD_#1
hac1_UD_#1
0.96 0.97 0.97 0.98 0.99 1.00
0.97 0.97 0.97 0.99 1.00 0.99
0.97 0.97 0.98 1.00 0.99 0.98
0.97 0.97 1.00 0.98 0.97 0.97
0.99 1.00 0.97 0.97 0.97 0.97
1.00 0.99 0.97 0.97 0.97 0.96
hac1_UD_#1
WT_UD_#1
WT_UD_#2
hac1_UD_#2
HDA6-OX2_UD_#2
HDA6-OX2_UD_#1
0.0 0.2 0.4 0.6 0.8 1.0
hac1_HD_#2
HDA6-OX2_HD_#1
HDA6-OX2_HD_#2
WT_HD_#1
WT_HD_#2
hac1_HD_#1
0.91 0.97 0.96 0.98 0.98 1.00
0.91 0.97 0.96 0.98 1.00 0.98
0.91 0.98 0.97 1.00 0.98 0.98
0.92 0.99 1.00 0.97 0.96 0.96
0.91 1.00 0.99 0.98 0.97 0.97
1.00 0.91 0.92 0.91 0.91 0.91
hac1_HD_#1
WT_HD_#2
WT_HD_#1
HDA6-OX2_HD_#2
HDA6-OX2_HD_#1
hac1_HD_#2
0.0 0.2 0.4 0.6 0.8 1.0

Figure S2

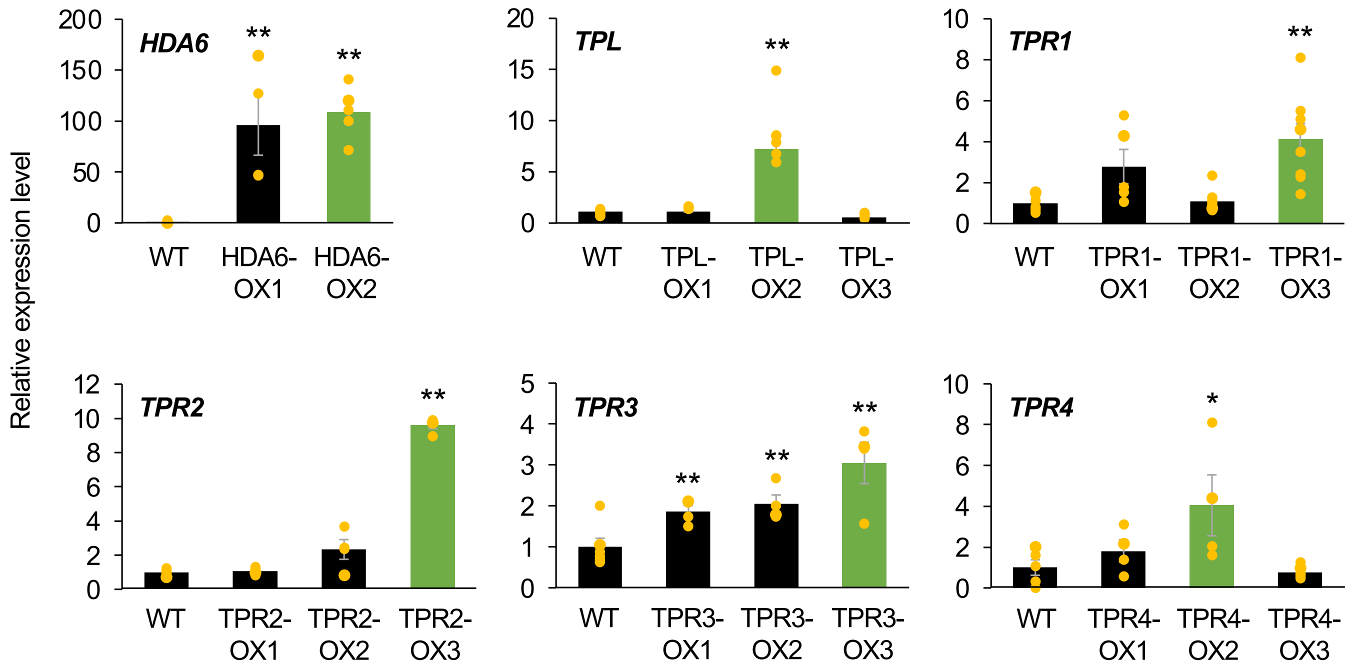
Relative expression level
HDA6
200
150
100
50
0
**
**
WT
HDA6-OX1
HDA6-OX2
TPL
20
15
10
5
0
**
WT
TPL-OX1
TPL-OX2
TPL-OX3
TPR1
10
8
6
4
2
0
**
WT
TPR1-OX1
TPR1-OX2
TPR1-OX3
TPR2
12
10
8
6
4
2
0
**
WT
TPR2-OX1
TPR2-OX2
TPR2-OX3
TPR3
5
4
3
2
1
0
**
**
**
WT
TPR3-OX1
TPR3-OX2
TPR3-OX3
TPR4
10
8
6
4
2
0
*
WT
TPR4-OX1
TPR4-OX2
TPR4-OX3

Figure S3

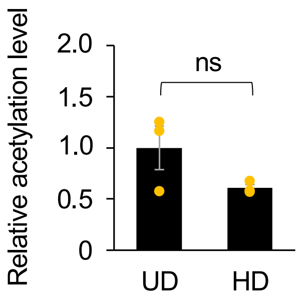

Figure S4

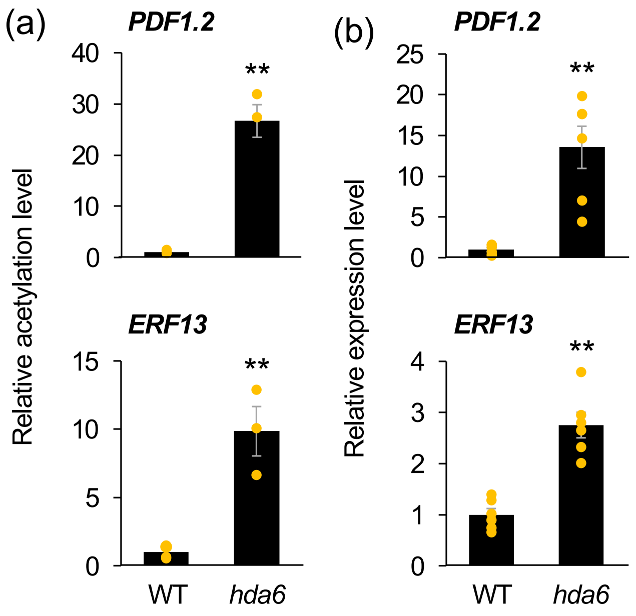

(a)
PDF1.2
40
30
20
10
0
**
ERF13
15
10
5
0
**
Relative acetylation level
WT
hda6
(b)
PDF1.2
25
20
15
10
5
0
**
ERF13
4
3
2
1
0
**
Relative expression level
WT
hda6

Figure S5

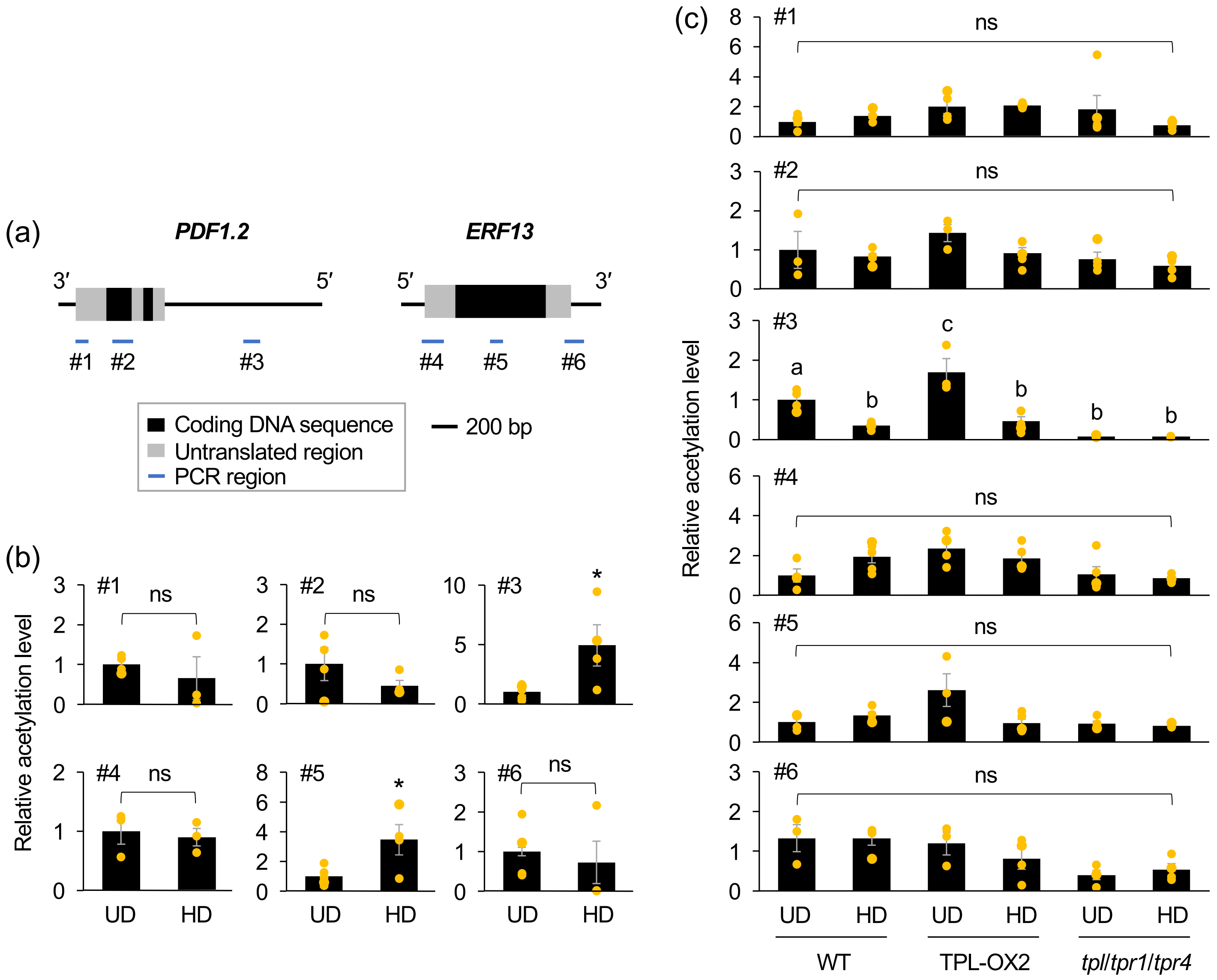
(a)
PDF1.2
ERF13
3′
5′
5′
3′
#1
#2
#3
#4
#5
#6
Coding DNA sequence
Untranslated region
PCR region
200 bp
(b)
Relative acetylation level
#1
ns
#2
ns
#3
*
#4
ns
#5
*
#6
ns
UD
HD
UD
HD
UD
HD
(c)
Relative acetylation level
#1
ns
#2
ns
#3
a
b
c
b
b
b
#4
ns
#5
ns
#6
ns
UD
HD
UD
HD
UD
HD
WT
TPL-OX2
tpl/tpr1/tpr4

Figure S6

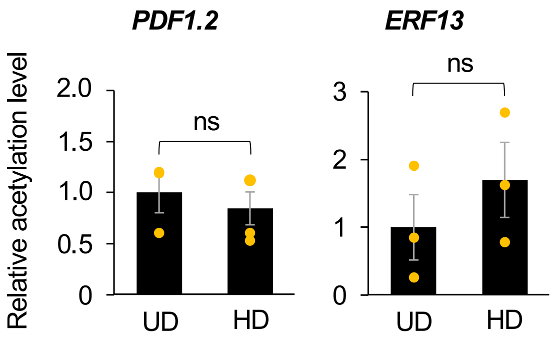
PDF1.2
ERF13
Relative acetylation level
2.0
1.5
1.0
0.5
0
ns
UD
HD
3
2
1
0
ns
UD
HD

Figure S7

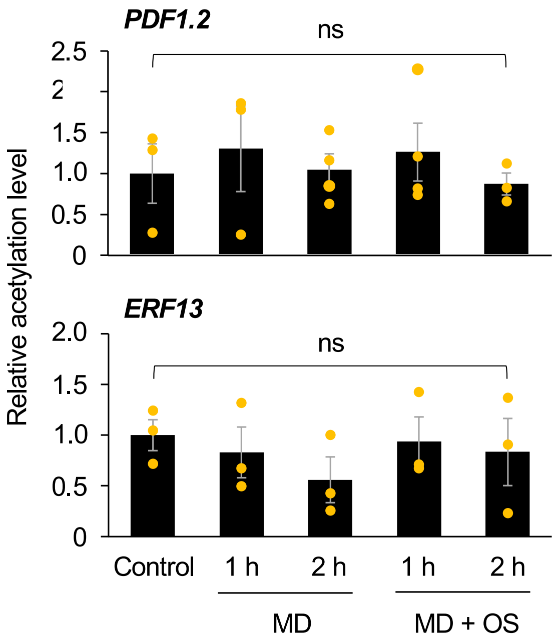
PDF1.2
ns
2.5
2
1.5
1.0
0.5
0
Relative acetylation level
ERF13
2.0
1.5
1.0
0.5
0
ns
Control
1 h
2 h
1 h
2 h
MD
MD + OS

Figure S8

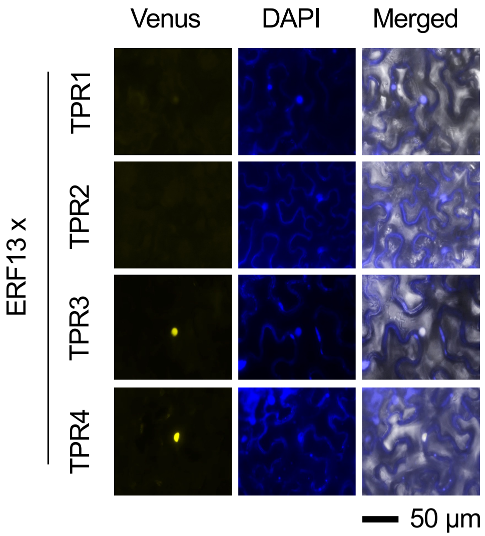

Figure S9

FIGURE S1 Detection of recombinant proteins. (a) Biotinylated HDA6 protein synthesized using the cell-free system and detected by immunoblotting with an anti-biotin antibody. (b) FLAG-conjugated proteins synthesized using the cell-free system and detected by immunoblotting with an anti-FLAG antibody. Predicted protein signals are indicated by arrowheads.

FIGURE S2 Reproducibility between two replicates in ChIP-Seq analysis. Spearman correlation coefficients were calculated between the read counts of two samples in ChIP-Seq analysis of histone H3 (a) and H3K9 acetylation (b).

FIGURE S3 Relative expression levels of the corresponding genes in mature leaves of Arabidopsis wild-type (WT) plants and overexpression (OX) lines of Arabidopsis plants transformed with *HDA6*, *TPL*, *TPR1*, *TPR2*, *TPR3*, or *TPR4*. The line used for additional experiments is indicated by green columns. Individual data points are shown with means and standard errors (n = 4–8). An asterisk(s) indicates significant differences compared to WT, as determined by ANOVA with Holm's sequential Bonferroni post hoc test (**, $p < 0.01$; *, $0.01 \leq p < 0.05$).

FIGURE S4 H3K9 acetylation levels on *PBL27* in leaves of undamaged (UD) wild-type plants and those in response to damage by *Spodoptera litura* larvae for 24 h (HD). Individual data points are shown with means and standard errors (n = 3). Data are not significantly different between UD and HD, as determined by Student's *t*-test (ns, $p \geq 0.05$).

FIGURE S5 H3K9 acetylation (a) and expression levels (b) on *PDF1.2* and *ERF13* in leaves of undamaged wild-type (WT) and *hda6* plants. Individual data points are shown with means and standard errors (n = 3–4 and n = 6 for a and b, respectively). An asterisk indicates significant difference compared to WT, as determined by Student's *t*-test (**, $p < 0.01$).

FIGURE S6 The loci of H3K9 acetylation within the promoter region and gene body of the *PDF1.2* and *ERF13*. (a) The position of the coding DNA sequences, the untranslated regions, and a series of polymerase chain reaction amplified regions in *PDF1.2* and *ERF13*. (b) H3K9 acetylation levels at *PDF1.2* and *ERF13* in undamaged (UD) wild-type (WT) and those in response to damage by *Spodoptera litura* larvae for 24 h (HD). (c) Immunoprecipitation of proteins in leaves of undamaged WT, TPL-OX2 and *tpl*/*tpr1*/*tpr4*

plants and those damaged by *Spodoptera litura* larvae for 2 h was performed using the anti-HDA6 antibody. The selected region at *PDF1.2* and *ERF13* bound by the immunoprecipitated HDA6 proteins were quantified. Individual data points are presented with mean values and standard errors (n = 3–5). For (b), an asterisk indicates significant difference compared to WT, as determined by Student's t-test (*, $0.01 \leq p < 0.05$). For (c), means indicated by different small letters are significantly different based on ANOVA with post hoc Tukey HSD ($p < 0.05$).

FIGURE S7 H3K9 acetylation levels in locally damaged areas (approximately 2 mm distal to the damage sites) of leaves of undamaged (UD) wild-type plants and those in response to damage by *Spodoptera litura* larvae for 1 h (HD). Individual data points are shown with means and standard errors (n = 3-4). Data are not significantly different between UD and HD plants, as determined by Student's t-test (ns, $p \geq 0.05$).

FIGURE S8 H3K9 acetylation levels in leaves following mechanical damage (MD) with application of *Spodoptera litura* oral secretion (OS). Wild-type Arabidopsis leaves were treated with MD or MD + OS for 1 or 2 h. Undamaged and untreated leaves were used as control. Individual data points are presented with mean values and standard errors (n = 3–4). Data are not significantly different from control, as determined by ANOVA (ns, $p \geq$ 0.05).

FIGURE S9 *In planta* interaction between ERF13 fused to the N-terminal fragment of Venus and each TPL/TPR protein fused to the C-terminal fragment of Venus in *Nicotiana benthamiana* leaf cells, using bimolecular fluorescence complementation analysis. The images show the reconstructed Venus signal, DAPI (4′,6-diamidino-2-phenylindole) fluorescence, and the merged image with bright field.
